# Potentiation of cord blood cell therapy with erythropoietin for children with CP: a 2 × 2 factorial randomized placebo-controlled trial

**DOI:** 10.1186/s13287-020-02020-y

**Published:** 2020-11-27

**Authors:** Kyunghoon Min, Mi Ri Suh, Kye Hee Cho, Wookyung Park, Myung Seo Kang, Su Jin Jang, Sang Heum Kim, Seonkyeong Rhie, Jee In Choi, Hyun-Jin Kim, Kwang Yul Cha, MinYoung Kim

**Affiliations:** 1 Department of Rehabilitation Medicine, CHA Bundang Medical Center, CHA University School of Medicine, 59 Yatap-ro, Bundang-gu, Seongnam, Gyeonggi-do Republic of Korea; 2 Rehabilitation and Regeneration Research Center, CHA University, Seongnam, Republic of Korea; 3 Department of Rehabilitation Medicine, CHA Ilsan Medical Center, CHA University School of Medicine, Ilsan, Republic of Korea; 4 Department of Laboratory Medicine, CHA Bundang Medical Center, CHA University School of Medicine and CHA Cord Blood Bank, Seongnam, Republic of Korea; 5 Department of Nuclear Medicine, CHA Bundang Medical Center, CHA University School of Medicine, Seongnam, Republic of Korea; 6 Department of Radiology, CHA Bundang Medical Center, CHA University School of Medicine, Seongnam, Republic of Korea; 7 Department of Pediatrics, CHA Bundang Medical Center, CHA University School of Medicine, Seongnam, Republic of Korea; 8 CHA Hollywood Presbyterian Medical Center, Los Angeles, CA USA

**Keywords:** Umbilical cord blood, Erythropoietin, Cerebral palsy, Clinical trial, Functional performance

## Abstract

**Background:**

Concomitant administration of allogeneic umbilical cord blood (UCB) infusion and erythropoietin (EPO) showed therapeutic efficacy in children with cerebral palsy (CP). However, no clinical studies have investigated the effects of UCB and EPO combination therapy using a 2 × 2 four-arm factorial blinded design with four arms. This randomized placebo-controlled trial aimed to identify the synergistic and individual efficacies of UCB cell and EPO for the treatment of CP.

**Methods:**

Children diagnosed with CP were randomly segregated into four groups: (A) UCB+EPO, (B) UCB+placebo EPO, (C) placebo UCB+EPO, and (D) placebo UCB+placebo EPO. Based on the UCB unit selection criteria of matching for ≥ 4/6 of human leukocyte antigen (HLA)-A, -B, and DRB1 and total nucleated cell (TNC) number of ≥ 3 × 10^7^/kg, allogeneic UCB was intravenously infused and 500 IU/kg human recombinant EPO was administered six times. Functional measurements, brain imaging studies, and electroencephalography were performed from baseline until 12 months post-treatment. Furthermore, adverse events were closely monitored.

**Results:**

Eighty-eight of 92 children enrolled (3.05 ± 1.22 years) completed the study. Change in gross motor performance measure (GMPM) was greater in group A than in group D at 1 month (△2.30 vs. △0.71, *P* = 0.025) and 12 months (△6.85 vs. △2.34, *P* = 0.018) post-treatment. GMPM change ratios were calculated to adjust motor function at the baseline. Group A showed a larger improvement in the GMPM change ratio at 1 month and 12 months post-treatment than group D. At 12 months post-treatment, the GMPM change ratios were in the order of groups A, B, C, and D. These results indicate synergistic effect of UCB and EPO combination better than each single therapy. In diffusion tensor imaging, the change ratio of fractional anisotropy at spinothalamic radiation was higher in group A than group D in subgroup of age ≥ 3 years. Additionally, higher TNC and more HLA-matched UCB units led to better gross motor outcomes in group A. Adverse events remained unchanged upon UCB or EPO administration.

**Conclusions:**

These results indicate that the efficacy of allogeneic UCB cell could be potentiated by EPO for neurological recovery in children with CP without harmful effects.

**Trial registration:**

ClinicalTrials.gov, NCT01991145, registered 25 November 2013.

## Background

Cerebral palsy (CP), the leading cause of motor impairment in early childhood, causes life-long disabilities [1, 2]. Clinical improvements following conventional rehabilitation or surgical therapies are limited [1]. Children with CP also present motor improvement to an extent until certain age [3]. Thereafter, it is difficult to acquire higher gross motor function and further functional decline may be observed in severely disabled children [4]. Lasting neuroinflammation and apoptosis occur in brains of CP patients, which cannot be corrected with conventional therapeutic approaches [5]. These disruptions influence the endogenous repair and regeneration after primary insult to the immature brain, known as a tertiary pathomechanism [6]. Cell and growth factor therapies are suggested to have therapeutic effects against this pathogenesis [6, 7].

Cell therapy in CP has been investigated for more than 10 years [1, 8]. The cell types used in clinical trials were umbilical cord blood (UCB) cells, olfactory ensheathing cells, neural stem cells, and neural progenitor cells [9]. Among these various cell types, the UCB containing stem cells are reportedly safe even for newborns [9–11]. Since its first use in 1988, UCB has been administered in over 100 indications including neurological disorders without reports of harmful effects [12–14]. UCB has been suggested to exert neuroprotective, anti-inflammatory, and anti-apoptotic effects [15]. Although autologous UCB may be ideal with positive results in previous clinical trials, most children with CP do not possess their own UCBs [16, 17]. UCB has substantial advantages over other cell sources because UCB has been banked worldwide and allogeneic UCB can be an alternative option with advantage of immune-tolerant characteristics [18].

So far, cell therapy has shown its efficacy mostly in preclinical stem cell researches. The main reasons that clinical applications of cell therapies for CP remain in the experimental stage are safety concerns and insufficient efficacy issues. Growth factors such as erythropoietin (EPO) and the granulocyte colony-stimulating factors have been introduced to potentiate the efficacy of cell therapy [19, 20]. EPO was reported to exert neuroprotective and neural repair effects, particularly in a neonatal hypoxic/ischemic brain injury CP model [21]. In a rat model of stroke, combination therapy with UCB cell and EPO exerted synergistic effects on neurological recovery, characterized by neurogenesis and angiogenesis, compared to UCB or EPO monotherapy [22]. Since both UCB and EPO could stimulate the same Akt signaling pathway, the effect of UCB might be reinforced by EPO [23, 24]. Furthermore, the clinical use of EPO showed neuroprotective effects among preterm infants [25, 26].

In our previous clinical trial, children with CP-administered intravenous allogeneic UCB infusion with EPO showed better outcomes than those administered EPO alone and control groups [27]. A subsequent trial assessing the therapeutic efficacy of UCB monotherapy suggested a therapeutic potential of UCB with its immunomodulatory characteristics including systemic pentraxin 3 (PTX3) upregulation [28]. However, the synergistic effect of UCB and EPO has not been assessed by direct group comparisons. This 2 × 2 factorial-designed double-blind placebo-controlled randomized trial was performed to identify the individual and/or synergistic efficacies of UCB and EPO combination therapy in children with CP for 1 year, with a longer period than that of our previous trials. In addition to the assessment of the functional changes, we assessed changes in the brain tissue through brain imaging and electroencephalography (EEG). Molecules potentially associated with neurological recovery were assayed and specific conditions of UCB and its recipients, serving as potential indicators of treatment effectiveness were also analyzed herein.

## Methods

### Participants

The inclusion criteria were children diagnosed with CP between 10 months and 6 years of age who had (i) allogeneic UCB units with criteria of ≥ 3 × 10^7^/kg total nucleated cell (TNC) number and matched for ≥ 4/6 of the human leukocyte antigen (HLA)-A, B, and DRB1 at high resolution and (ii) a hemoglobin level ≤ 13.6 g/dL. Parents or representatives provided written informed consent to participate in the study. The exclusion criteria were aspiration pneumonia, genetic diseases, hypersensitivity to the study medications, coagulopathy, intractable epilepsy, hypertension, hepatic or renal impairments, malignancies, and absolute neutrophil count ≤ 500/dL. The protocol was approved by the institutional review board (No. 2013-04-41) and the Korean Ministry of Food and Drug Safety (No. 12515) (Clinicaltrials.gov NCT01991145) [29].

### Study design and masking

**Fig. 1:**
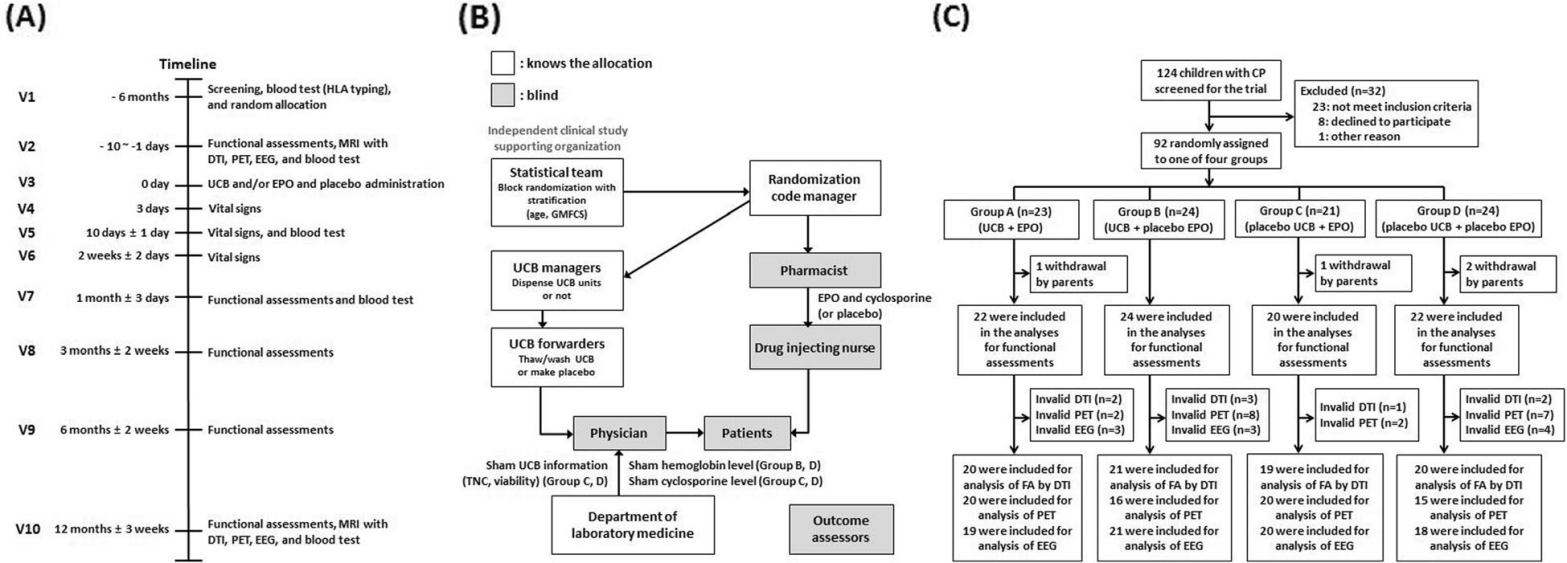
Screening, randomization, and follow-up. **a** The timeline of the study, **b** the cooperation of investigators to maintain double-blindness, and **c** the study flow. CP, cerebral palsy; DTI, diffusion tensor image; EEG, electroencephalogram; EPO, erythropoietin; FA, fractional anisotropy; GMFCS, gross motor function classification system; HLA, human leukocyte antigen; MRI, magnetic resonance imaging; PET, positron emission tomography; UCB, umbilical cord blood

The procedure was conducted as a double-blind placebo-controlled randomized trial. Participants were assigned into four groups using a block randomization code generated with SAS version 9.2 (SAS Institute Inc., Cary, NC, USA): (A) UCB+EPO, (B) UCB+placebo EPO (P-EPO), (C) placebo UCB (P-UCB)+EPO, and (D) P-UCB+P-EPO. Randomization was stratified by 2 factors: age (< 3 vs ≥ 3 years) and severity in the gross motor function classification system (GMFCS) level (GMFCS I–III, vs GMFCS IV–V) to ensure an even distribution into the allocation arms. The sample size was planned to recruit 30 patients per each group, total number of 120, based on central limit theorem [29]. To maintain blindness of the study of all participants, researchers, and outcome assessors to the treatment, an elaborate cooperation protocol was used (Fig. 1) [29]. Placebo materials of UCB, EPO, and cyclosporine were used. P-UCB was made from the subject’s own peripheral blood by UCB managers on the day of UCB therapy with the same appearance of UCB. Laboratory results such as the levels of hemoglobin affected by EPO and cyclosporine in the placebo groups which may affect the blindness of investigators were given artificial values by a designated investigator in the Department of Laboratory Medicine. The sham results were replaced by true values after completion of the study.

All data were recorded on government-sponsored online case reporting system using the internet-based Clinical Research and Trial management system, Korea (C140005), and managed independently.

### Procedures

Allogeneic UCB units were selected from the affiliated CHA cord blood bank after approval of Korean Organ Sharing Center. ABO blood types were matched, and two units of UCB were allowed to maintain the cell dose. Before administration, each unit was washed to eliminate dimethyl sulfoxide [30]. A single intravenous infusion of UCB or its placebo was performed. Groups A and B were administered with oral cyclosporine (ChongKunDang Pharm, Corp., Korea) at a dose of 7 mg/kg bid per day starting from 3 days before UCB administration; the same prescription was continued for 16 days (D− 3 to D+ 12 days). Groups C and D were administered placebos of UCB prepared from autologous peripheral blood and cyclosporine vehicle.

All participants in group A and C were administered EPO (Espogen®, LG Chem, Ltd., Korea) intravenously at a dose of 500 IU/kg at 2 h before UCB or placebo infusion. Subsequently, from D+ 3, each subject was injected five additional times with EPO at the same dose subcutaneously at 3-day intervals. Groups B and D were administered the EPO vehicle as a placebo. The vehicle placebo cyclosporine and EPO were provided by their own pharmaceutical companies.

All participants continued their conventional rehabilitation and were monitored for adverse events (AEs) (Fig. 1).

### Outcomes

#### Functional outcomes

Primary outcomes were the total scores of the gross motor performance measure (GMPM) [31], gross motor functional measure (GMFM) [32], and raw scores of mental and motor scales of the Bayley Scales for Infant Development-II (BSID-II) [33] which were assessed at baseline and 1, 3, 6, and 12 months after treatment (Additional file 1). The reliabilities of the primary outcomes among assessors were established by the clinical study team [34–36].

Subgroup analyses were conducted to estimate favorable indications for treatment according to the following clinical conditions: gestational age (GA) on birth divided as term (GA ≥ 37 weeks) vs preterm (GA < 37 weeks); severity in the motor function impairment divided as mild (GMFCS levels I–III) vs severe (GMFCS levels IV–V) impairment; and age at the time of the procedure divided as younger (< 3 years) vs older (≥ 3 years) ages.

Secondary outcome measures were other functional measures including GMFCS [37], Pediatric Evaluation of Disability Inventory [38], Functional Independence Measure for Children [39], summed scores on muscular strength by Medical Research Council scale [40], Beery-Buktenica developmental test of visual-motor integration [41], selective control assessment of lower extremity [42], modified Ashworth scale [43], modified Tardieu scale [44], and Quality of Upper Extremity Skills Test [45] (Additional file 2). All functional outcomes were assessed as planned in the trial protocol by trained assessors who were not aware of group assignment.

#### Survey of parent perception of the intervention

The subjective satisfaction towards the intervention was surveyed among the caregivers of the patients at completion of the study before the group allocation was open (Additional file 2).

#### Imaging studies and electroencephalogram (EEG)

Brain magnetic resonance imaging (MRI) and ^18^F-fluorodeoxyglucose positron emission tomography/computed tomography (^18^F-PET/CT) images were acquired at baseline and at 12 months after intervention. Diffusion tensor imaging (DTI) data from brain MRI were obtained to determine the effects of treatment on the white matter integration. Fractional anisotropy (FA) values were calculated by a voxel-based approach using the Tract-Based Spatial Statistics tool in an automated process [46, 47]. There are a total of 17 different white-mater tracts—single corpus callosum and bilateral fibers of eight tracts such as the anterior thalamic radiation (ATR), the cingulum in the cingulate cortex area, the cingulum in the hippocampal area, the corticospinal tract, the inferior fronto-occipital fasciculus, the superior and the inferior longitudinal fasciculus, and the uncinate fasciculus (Additional file 3)—from JHU white matter tractography atlases [48]. ^18^F-PET/CT images were acquired to assess differences in the regional brain glucose metabolism between groups and between the pre-treatment and the post-treatment imaging data (Additional file 4). Furthermore, sleeping asleep EEG was performed at baseline and 12 months after treatment. The average delta/alpha band power ratio (DAR) was obtained from five brain regions including the frontal, central, temporal, parietal, and occipital cortices, and their differences from pre-treatment to 12 months post-treatment were determined (Additional file 5).

#### Cytokines

Cytokines were analyzed using blood samples collected at 4 days before UCB infusion (D− 4), at the day of UCB injection prior to infusion (D− 0), and at 3 days, 10 days, and 30 days after UCB infusion (D+ 3, D+ 10, and D+ 30). Plasma levels of PTX-3, IL-8, TNF-α, and IL-1β were measured by an enzyme-linked immunosorbent assay and mRNA expression of the corresponding cytokines was measured by the reverse transcription polymerase chain reaction (Additional file 6) [28].

### Statistical analyses

Statistical analyses were performed using SPSS version 21.0 software (SPSS, Inc., Chicago, IL, USA) and Prism 5.0 software (GraphPad, Inc., San Diego, CA, USA). Categorical variables were analyzed by the Fisher’s exact test. Functional outcomes and the FA values from DTIs were compared by Kruskal-Wallis test with post hoc analyses and Mann-Whitney *U* test appropriately. As for primary outcomes (GMPM and GMFM), changes in raw scores from baseline were compared among four groups at each time point (1, 3, 6, and 12 months). Then, the changed values between baseline and each time point were divided by baseline values, expressed as GMPM or GMFM change ratio in order to adjust the baseline function. Ratio values were also compared as changes of raw scores.

Analysis of variance (ANOVA) and the paired *t* test were used to evaluate regional brain glucose metabolism. EEG data were analyzed with the iSyncBrain® software version 2.0 (iMediSync, Inc., Seoul, Korea). Average DARs were calculated from five brain regions and the Mann-Whitney *U* test was used. Data were locked on March 27, 2018, and all statistical analyses were confirmed by a statistician. Missing data were filled in by the last observational carried forward imputation.

## Results

From December 2013 to May 2016, 124 children with CP were enrolled, and 32 were excluded. Ninety-two subjects were randomly assigned to each group and four subjects withdrew their participation after the randomization. Eighty-eight participants (3.05 ± 1.22 years) were finally included: group A (UCB+EPO, *n* = 22), group B (UCB+P-EPO, *n* = 24), group C (P-UCB+EPO, *n* = 20), and group D (P-UCB+P-EPO, *n* = 22) (Fig. 1, Additional file 7). The demographic data revealed no significant differences in baseline variables among the groups (Table 1).

**Table 1:** Demographic and baseline participant characteristics (*n* = 88)

Group^a^	Group A (*n* = 22)	Group B (*n* = 24)	Group C (*n* = 20)	Group D (*n* = 22)
**Demographics**				
Sex, no. % male	10 (45.5%)	11 (45.8%)	10 (50.0%)	15 (68.2%)
Age, year; mean (SD; range)^b^	3.0 (1.2; 1.5–6.3)	2.9 (1.3; 1.0–5.0)	3.4 (1.3; 1.1–5.8)	3.0 (1.1; 1.2–6.0)
Gestational age, weeks; mean (SD; range)	32.3 (4.8; 26–41)	31.9 (3.9; 26–40)	31.9 (4.3; 26–40)	33.6 (5.4; 24–42)
Preterm, no. (%)	16 (72.7%)	20 (83.3%)	16 (80.0%)	13 (59.1%)
Birth weight (SD; range), kg	1.9 (.8; .6–3.6)	1.9 (.8; .8–3.4)	1.9 (.8; .7–3.5)	2.2 (.9; .7–4.2)
NBW/LBW/VLBW/ELBW^c^	6/7/8/1	5/10/7/2	5/8/5/2	10/7/3/2
GMFCS (I/II/III/IV/V)	1/2/5/6/8	2/2/5/3/12	1/6/3/7/3	0/1/5/10/6
Typology (SB/SU/D/C/A)^d^	18/0/3/0/1	20/0/4/0/0	15/0/4/0/1	17/0/4/0/1
**Baseline primary outcome measures**				
GMFM	38.0 (22.9)	31.9 (24.4)	44.3 (21.8)	31.1 (16.2)
GMPM	34.1 (14.7)	32.7 (13.6)	38.1 (11.5)	35.9 (11.2)
BSID-II mental raw score	106.4 (38.5)	99.2 (44.1)	121.7 (33.2)	100.9 (39.1)
BSID-II motor raw score	49.8 (19.9)	48.3 (24.5)	61.1 (20.1)	47.5 (21.5)
**MRI finding s**[49]				
Normal (*n* = 0)	0	0	0	0
Acquired lesions (*n* = 87)				
Periventricular leukomalacia (*n* = 66)	17	20	14	15
Diffuse encephalopathy (*n* = 18)	4	4	5	5
Focal ischemia/hemorrhage (*n* = 1)	0	0	0	1
Multicystic encephalomalacia (*n* = 2)	1	0	0	1
Malformations (*n* = 0)				
Cortical dysplasia (*n* = 0)	0	0	0	0
Schizencephaly (*n* = 0)	0	0	0	0
Corpus callosum agenesis (*n* = 0)	0	0	0	0
Miscellaneous/unknown (*n* = 1)				
Miscellaneous etiologies (*n* = 0)	0	0	0	0
Abnormality of white matter signal (*n* = 1)	0	0	1	0

Values represent number of patients unless otherwise noted. No baseline characteristics were significantly different among four groups (*P* value > 0.05 for all comparisons). Baseline primary outcome measures are shown as means (SD)

^a^Group A (*n* = 22) received UCB and EPO, group B (*n* = 24) received UCB and placebo EPO, group C (*n* = 20) received placebo UCB and EPO, and group D (*n* = 22) received placebo UCB and placebo EPO

^b^Age at the time of intervention, corrected for preterm birth

^c^NBW was defined as birth body weight ≥ 2500 g, LBW < 2500 g, VLBW < 1500 g, and ELBW < 1000 g

^d^Typology was divided as follows: SB, SU, D, C, and A

Abbreviations: Birth weight (*NBW* normal birth weight, *LBW* low birth weight, *VLBW* very low birth weight, *ELBW* extremely low birth weight), *BSID-II* Bayley scales of infant development-II, *EPO* erythropoietin, *GMFM* gross motor function measure, *GMPM* gross motor performance measure, Typology (*SB* spastic bilateral, *SU* spastic unilateral, *D* dystonic, *C* choreoathetoid, *A* ataxic), *UCB* umbilical cord blood

### Adverse events

In groups A and C who were administered true EPO, the levels of hemoglobin, hematocrit, and red blood cells increased to the upper reference limits at 1 month post-therapy and then returned to the baseline levels (Additional file 8). All other laboratory data were within the reference ranges during the study period.

Eleven serious AEs were reported in the safety set. The distributions of serious AEs and non-serious AEs did not differ among the four groups, and all subjects recovered (Additional file 9).

### Functional outcomes

**Fig. 2:**
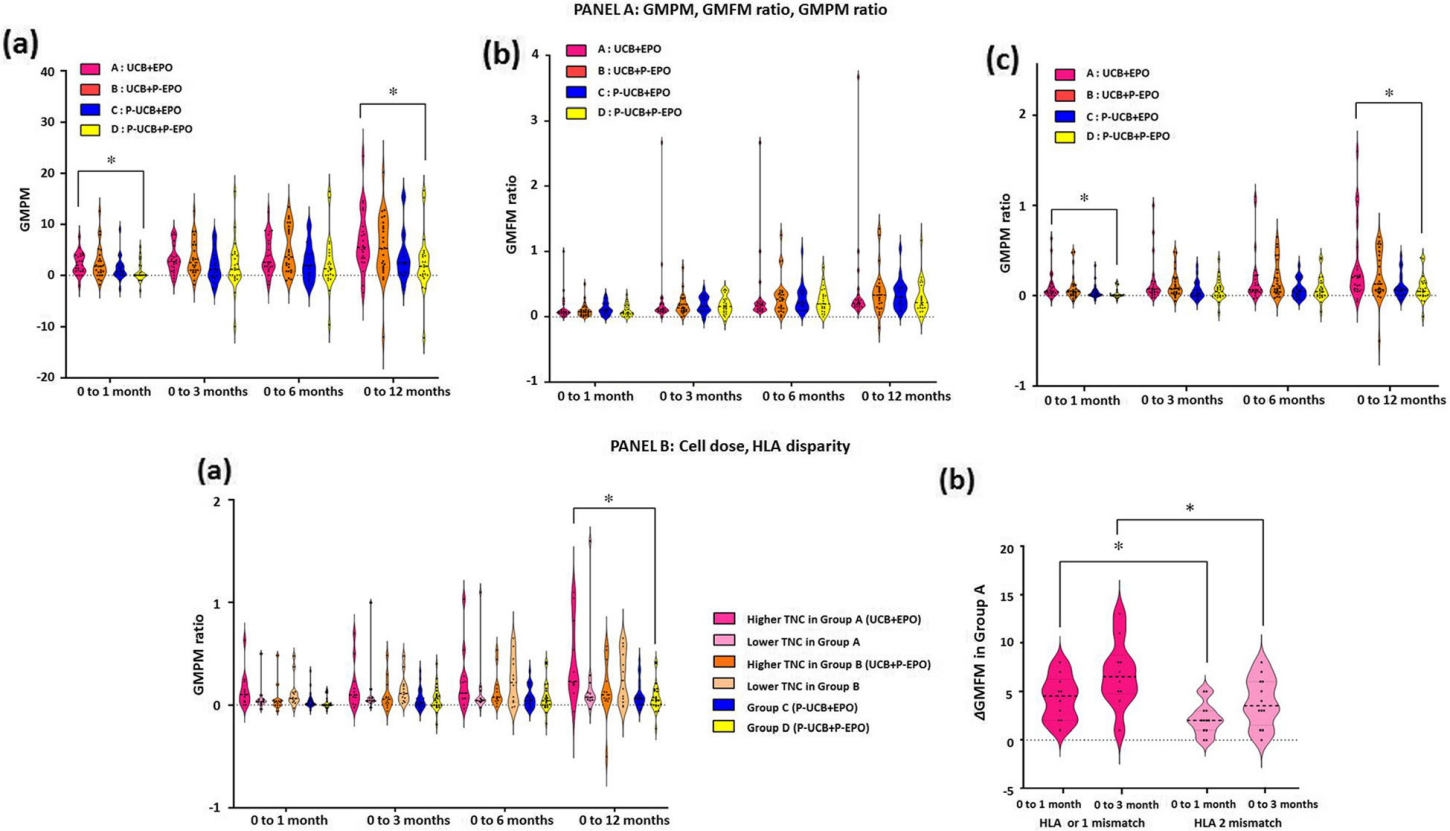
Changes in gross motor outcome. **A** Changes in (a) GMPM, (b) GMFM change ratio, and (c) GMPM change ratio from baseline to 1, 3, 6, and 12 months post-treatment among group A, B, C, and D. GMPM and GMFM change ratios were calculated as $\frac{\left(\mathrm{GMPM}\ \mathrm{at}\ \mathrm{the}\ \mathrm{time}\ \mathrm{point}-\mathrm{GMPM}\ \mathrm{at}\ \mathrm{baseline}\ \right)}{\mathrm{GMPM}\ \mathrm{at}\ \mathrm{baseline}\ }$ and $\frac{\left(\mathrm{GMFM}\ \mathrm{at}\ \mathrm{the}\ \mathrm{time}\ \mathrm{point}-\mathrm{GMFM}\ \mathrm{at}\ \mathrm{baseline}\ \right)}{\mathrm{GMFM}\ \mathrm{at}\ \mathrm{baseline}\ }$, respectively. Group A (*n* = 22) received umbilical cord blood (UCB) with erythropoietin (EPO), group B (*n* = 24) received UCB with placebo EPO (P-EPO), group C (*n* = 20) received placebo UCB (P-UCB) and EPO, and group D (*n* = 22) received P-UCB and P-EPO. Data are shown in violin plots where dots represent each value, bold dotted lines represent the median and fine dotted lines represent lower and upper quartiles. Asterisk indicates significant difference in outcome scores between two groups based on post hoc analyses (*P* < 0.05) (Dunn’s multiple comparison test) following Kruskal-Wallis test. **B** Changes in GMPM change ratio according to (a) cell dose and (b) HLA disparity in group A. Subgroups with lower and higher TNC were categorized according to the median value of TNC in groups A and B. Subgroup from group A with higher TNC showed significant improvement in GMPM change ratio compared to group D after 12 months post-intervention. Data are also shown in violin plots where dots represent each value, bold dotted lines represent the median and fine dotted lines represent lower and upper quartiles. Asterisk indicates significant difference in outcome scores between two groups based on post hoc analyses (*P* < 0.05) (Dunn’s multiple comparison test) following Kruskal-Wallis test. The impact of HLA incompatibility was analyzed between HLA full-matched or 1 mis-matched and HLA 2 mis-matched cases in group A and B. In group A, variances of GMFM during baseline to 1 month (*P* = 0.036) and to 3 months (*P* = 0.05) were larger among the subjects who received more HLA-compatible UCB (*n* = 10) than those treated with HLA 2-mismatched UCB (*n* = 12). Asterisk indicates significant difference in outcome scores between two groups based on Mann-Whitney *U* test. EPO, erythropoietin; GMFM, gross motor function measure; GMPM, gross motor performance measure; HLA, human leukocyte antigen; TNC, total nucleated cell; UCB, umbilical cord blood

There were no significant differences in baseline measurements among the four groups. All groups showed improvements in primary outcomes except for GMPM in group D during 1 year. Group A showed a greater improvement in the GMPM score at 1 month (△2.30) and 12 months (△6.85) post-treatment compared to group D (△0.71 and △2.34) (*P* = 0.025 and *P* = 0.018, respectively) (Fig. 2A (a), Additional file 10). Randomization was stratified according to motor severity and age at the baseline, likely explaining the reason of the functional status that did not differ among the four groups. Despite performing a stratified randomization to ensure an even distribution, more participants in group C tended to have better motor function. Thus, we also calculated GMPM change ratios as ($\frac{\left(\mathrm{score}\ \mathrm{at}\ \mathrm{the}\ \mathrm{time}\ \mathrm{point}-\mathrm{score}\ \mathrm{at}\ \mathrm{baseline}\ \right)}{\mathrm{score}\ \mathrm{at}\ \mathrm{baseline}\ }$) for outcome comparisons to adjust motor function at the baseline. Group A showed a larger improvement in the GMPM change ratio at 1 month (0.11) and 12 months (0.33) post-treatment than group D (0.02 and 0.07) (*P* = 0.023 and *P* = 0.016, respectively) (Fig. 2A (c), Additional file 11). At 12 months post-treatment, the GMPM change ratios were in the order of groups A, B, C, and D, with changes in the GMFM ratio showing the same order (Fig. 2A (b, c)). These results indicate synergistic effect from UCB and EPO combination according to the changed score value in comparison with those values in individual therapies. The improved GMPM score (Δ 6.85) of group A is higher than those of group B (Δ 5.58) or C (Δ 3.67) at 12 months post-treatment.

Efficacy factor analysis for UCB conditions revealed two significant findings (Additional file 12). When participants in groups A and B were divided into 2 subgroups by the median TNC value per body weight of each groups, the higher TNC subgroup in group A than in group D resulted in greater improvement in the GMPM change ratio at 12 months post-treatment (Fig. 2B (a)). Additionally, subjects administered higher matched units (HLA full-matched or 1 mis-matched; *n* = 10) showed greater increases in the GMFM score than those administered with the HLA 2 mis-matched units (*n* = 12) in group A at 1 month and 3 months post-treatment (*P* = 0.036 and *P* = 0.05, respectively) (Fig. 2B (b)). The changes of BSID-II raw scores in four groups were not different during the study period. Other secondary outcomes also did not differ among four groups.

### Survey of parent perception of the intervention

The survey among the caregivers showed significantly higher satisfaction for language improvement in group A (*P* = 0.05) and for mental improvement in group B (*P* = 0.015) compared to those in group D (Additional file 13).

### Subgroup analyses

#### Mild vs severe impairment

In the severe impairment subgroup (*n* = 55), group A showed a greater improvement in the GMPM change ratio compared to groups C and D, whereas comparison in the mild impairment group (*n* = 33) did not show a different outcome (panel A in Additional file 14).

#### Term vs preterm

In the term birth subgroup (*n* = 23), groups A and B showed a greater improvement in the GMPM change ratio compared to that in the groups C and D. There were no significant differences among 4 groups in preterm birth subgroup (*n* = 65) (panel B in Additional file 14).

#### Younger vs older age

There were no significant differences on any outcome measures in neither younger (*n* = 37) or older (*n* = 51) subgroups.

### Structural changes in DTI

DTI data were obtained from 80 patients. No significant differences were observed in the FA change ratios calculated as ($\frac{\left(\mathrm{FA}\ \mathrm{at}\ \mathrm{the}\ \mathrm{time}\ \mathrm{point}-\mathrm{FA}\ \mathrm{at}\ \mathrm{baseline}\ \right)}{\mathrm{FA}\ \mathrm{at}\ \mathrm{baseline}\ }$) in 19 regions of interest among the 4 groups. However, in subpopulations of > 3 years, group A displayed the higher increment in the FA change ratio at the right ATR than group D (*P* < 0.05) (Additional file 15).

### Metabolic changes in PET/CT

PET/CT data from 71 patients were available for analysis. Increased glucose metabolism was observed at the bilateral cerebellar hemisphere in group B, whereas it was increased at the midbrain and the thalamus in group D (Additional file 16).

### EEG mapping of band power

**Fig. 3:**
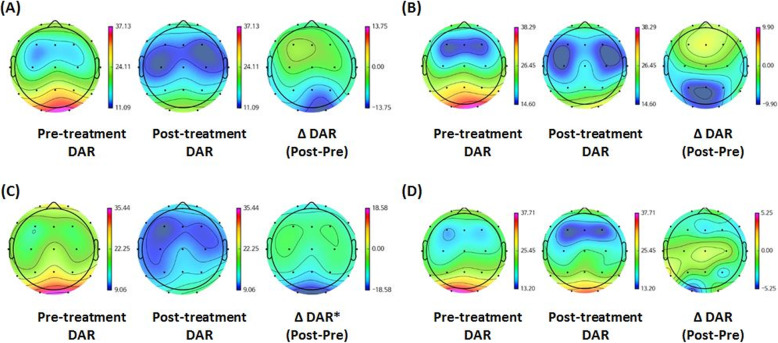
Electroencephalogram mapping before and after UCB injection. Average delta/alpha band power ratio (DAR) from electroencephalogram (EEG) is depicted on the 2D brain topomap. DAR from EEG taken before treatment, 12 months after treatment, and their difference (post-treatment–pre-treatment) are shown from left towards right. **a** Taken from group A (*n* = 20, mean age of pre-treatment EEG was 2.95 ± 1.20 years), **b** from group B (*n* = 20, mean age of pre-treatment EEG was 2.71 ± 1.27 years), **c** from group C (*n* = 20, mean age of pre-treatment EEG was 3.28 ± 1.27 years), and **d** from group D (*n* = 19, mean age of pre-treatment EEG was 4.17 ± 1.41 years). Among the total 88 participants, only 79 EEG data at baseline and 12 months post-treatment were able to be appropriately processed. Six participants lacked follow-up study, and 3 files were invalid on the analyzing program. *P < 0.05 by Mann-Whitney *U* test comparing the difference between pre- and post-treatment DAR. DAR, delta/alpha ratio; EEG, electroencephalogram; UCB, umbilical cord blood

EEG data from 78 patients were available for analysis. The relative value of average DAR showed a decreasing trend after treatment in groups A, B, and C, particularly at the posterior parietal and the occipital regions compared to that in group D. However, only group C showed a significant change between the baseline and post-treatment DAR (Fig. 3).

### Changes in mRNA expressions and cytokines associated with inflammation and innate immunity

Blood samples from 32 patients were available for analysis. They were re-grouped into 2 subgroups: those showing improvements in GMFCS (*n* = 12) or without improvements (*n* = 20) at 12 months post-treatment. The mRNA levels of IL-1*β* showed a greater increase at 3 days (*P* = 0.036) and 10 days (*P* = 0.013) post-treatment from baseline in the improved subgroup (Additional file 17).

In group B, when the cytokine change ratios calculated as ($\frac{\left(\mathrm{value}\ \mathrm{at}\ \mathrm{the}\ \mathrm{time}\ \mathrm{point}-\mathrm{value}\ \mathrm{at}\ \mathrm{baseline}\ \right)}{\mathrm{value}\ \mathrm{at}\ \mathrm{baseline}\ }$) were compared between “more-improved” and “less-improved” changes in the median GMPM score over 12 months, those of IL-8 and PTX3 were higher at 10 days post-treatment in the “more-improved” group than in the “less-improved” group (*P* = 0.039 and *P* = 0.031 respectively) (Additional file 18).

## Discussion

This study aimed to verify the results of our previous clinical trial regarding the efficacy and safety of UCB therapy potentiated with EPO in children with CP [27]. The therapeutic effect of allogeneic UCB combined with EPO on motor function was reproduced. Our two published trials of UCB plus EPO and UCB alone were incomplete to assess the efficacy of allogeneic UCB and/or EPO under the same controlled conditions. This is the first study to analyze the contributions of combined or individual UCB and EPO in children with CP among four groups.

We observed no harmful effects related to UCB, EPO, or their combination which were decided according to the statistical analysis, the period of the occurrence, likely risk of each serious event depending on the treatment the patients received. Immunosuppressant administration for 16 days did not increase the occurrence of AEs in groups A and B. In the previous trial, pneumonia and irritability occurred more in the UCB- and the EPO-administered groups, possibly because of the long 1-month duration of immunosuppression treatment [27]. In this study, pneumonia listed in non-serious adverse events seemed to be more noticeable in group C (4 cases) than the other groups (*P* = 0.058). However, they were decided to be unlikely related to the intervention, according to clinical context. Furthermore, among the four, two patients were reported to have pneumonia at the baseline screening period and not after the intervention. All participants could be followed for 3–5 years, and there were no reports of serious AEs suspected to be related to the treatment. As hypothesized, the levels of hemoglobin, hematocrit, and red blood cell counts were increased by EPO administration, which returned to baseline within 1 year; no thromboembolic events were observed.

In the analyses of efficacy, group A showed better outcomes in the GMPM and GMPM change ratio than group D (Δ6.85 vs. Δ2.34, and 0.33 vs. 0.04, respectively), at 12 months post-treatment (Fig. 2A; Additional files 10, 11). Additionally, the GMFM change ratio showed a similar trend without statistical significance. The GMFM and the GMPM are specific tools for evaluating gross motor ability in children with CP. Typically, the scores of GMFM and GMPM are highly correlated [34]. The GMFM represents motor function related to ambulatory ability, whereas GMPM assesses the quality of movement which is specifically applied to CP [31, 50]. Thus, UCB and EPO combination therapy improved gross motor ability without reaching alteration of ambulatory function. As shown in Fig. 2A, UCB mono- (group B) or EPO mono- (group C) therapy groups also showed the trend of better motor recovery than control group. However, only merging treatment of UCB and EPO (group A) demonstrated noteworthy improvement after 1 year. Considering the difficulties in gaining function and frequent occurrence of motor deterioration in CP, this finding may be clinically applicable [3]. Therefore, in future trials, repeated treatments with UCB and EPO combination may lead to greater functional improvements. For BSID-II, there were no differences between groups whereas it showed significant difference in our previous trial [27]. The reason could be thought as follows: First, the means of cell dose were lower (in this study, A group 4.8 × 10^7^, B group 5.0 × 10^7^) than that of our previous study (8.33 × 10^7^). Second, BSID-II may not have been able to reflect the changes because of its modest stability as a development assessment tool [51]. BSID-II has concerns on having limited floors and ceilings with selecting item sets. Third, there could be differences in clinical characteristics including typology and severity between the trials. Another prior clinical study also did not show significant findings in BSID-II [28]. However, evaluation tools seem to show different sensitivities according to slightly different typology and severity of study populations each time, and the tools which showed significance at 0–1 month seem to show consistent significance at longer terms [27, 28].

The differences in the number of introduced UCB cells in the clinical studies seemed to bring different results. In our previous trial, which used higher dose of the cells, GMPM score showed significant improvement by UCB and EPO therapy from 3 months [27], while it showed significance only at 12 months in this trial with small number of cells. Amount of cell dose is thought to be an important factor in administrating UCB as it was appeared in the previous clinical researches [17, 27, 28] and also an animal study showed a dose-response relationship [52]. Shorter duration of immunosuppression (16 days) in this trial than in the previous one (28 days) could be another factor. However, SAE and AE that might relate to use of cyclosporin was not reported this time. To enhance efficacy, administration of higher cell dosage and also repeated cell delivery could be suggested referring the other clinical trials [53, 54].

According to subgroup analyses, this therapy may be more effective in the severely motor impaired and term birth subgroup with a high risk of postnatal asphyxia. A higher cell dose and higher histocompatibility were reportedly found to be related to efficacy [27, 28]. First, TNC affected the treatment outcomes by increasing the GMPM score variance ratio in the higher TNC subgroup in group A, leading to better outcomes compared to those in groups C and D. Second, in terms of histocompatibility, fully matched and HLA 1-mismatched units administered to the subjects yielded better motor outcomes than those in the HLA 2-mismatched group. Thus, autologous UCB, although not available in most cases, may have superior clinical results in CP [17, 55].

Along with positive results upon functional measurements, brain imaging and EEG yielded certain significant findings. Analyses of DTI revealed the largest increase in the FA value in the right ATR in group A among patients of older ages (≥ 3 years), demonstrating improvement in the integrity of the white matter tract including myelination [56]. Changes in ATR indicate that facilitated reorganization occurred at the ascending somatosensory tracts [57]. The average age of group A was 3 years; at this age, the FA value does not typically increase, as the DTI values start plateauing at age 24 months [58, 59]. According to ^18^F-fluorodeoxyglucose-PET analysis, specific changes were not observed. In our previous studies with 2-week interval follow-up, inflammation was ameliorated in the posterior white matter [27, 28]. The large difference in the evaluation interval, which was 1 year in this study, appeared to give different results.

Brain wave analysis revealed a decreasing trend in DAR in groups A–C in the posterior cerebral cortices, whereas group D did not show this trend. While the delta band decrease starts from 6 months to 15 months [60] concurrent with the decreasing delta/theta band power ratio [61], alpha bands consistently increase with age showing over 80% dominant frequency within the alpha range by 3 years [62]. Therefore, the decreased DAR can be interpreted as the emergence of a more mature type of cortical sleep rhythms in the EEG.

Our previous study revealed increased PTX3 and IL-8 plasma levels within 2 weeks, which were correlated with functional outcomes in children with CP treated with UCB [28]. The current study also demonstrated the same results as plasma PTX3 and IL-8 levels were elevated in the more-improved subgroup only in group B. Thus, PTX3 and IL-8 appear to be related to the efficacy of UCB monotherapy. IL-8-mediated angiogenic pathway was known to be stimulated by UCB mononuclear cells [63]. Furthermore, IL-1β gene expression was elevated at 3 days and 10 days post-treatment in patients showing a definite improvement in the ambulatory level in groups A and B. IL-1β was known to be pro-inflammatory, but its neuroprotective characteristics in the injured brain was reported [64]. The core mechanism of the synergistic effect of UCB and EPO remains to be solved and the common pathway of UCB and EPO has not been investigated. IL-1β was increased in the subjects who are presumed to be responders in both groups A and B. And this new finding suggests potential role of IL-1β in neuroprotective mechanism of UCB and/or EPO treatment. Further studies will be required to determine other mechanisms other than those by PTX3 and IL-8.

This trial had some limitations. First, the ratio of outcome variance from baseline was adopted because subjects in group C tended to have better motor function. Although differences in baseline were not statistically significant, only a little difference may have affected their outcomes since it is more difficult to gain motor improvement from their palsied status in case of more severely impaired subjects [3, 4]. Therefore, we adopted changed ratios in the scores to minimize influence in their outcome by the baseline function. The efficacy showing time points in GMPM were 1 month and 12 months post-treatment, which differed from those in our previous studies, 3 and 6 months [27, 28]. This may be related to the lower cell number and shorter duration of immunosuppression in this study. Additionally, the ceiling effect may have also led to the negative results in Bayley scales. Lastly, the results subgroup analysis about cell dose and HLA disparity should be interpreted with deliberation due to small patient numbers.

## Conclusion

In conclusion, these results suggest that allogeneic UCB infusion therapy with EPO is safe and UCB plus EPO can be synergistically effective than single treatment of each for children with CP. More compatible and greater numbers of cells may lead to better outcomes. Further studies are necessary to reveal the core pathway related to neuronal recovery and means for potentiating this efficacy.

## Supplementary Information

**Additional file 1.** Primary functional outcome measurements.

**Additional file 2.** Secondary functional outcome measurements and survey of parent perception of the intervention.

**Additional file 3.** Brain MRI measurements and processing procedures.

**Additional file 4.** PET/CT measurements and processing procedures.

**Additional file 5.** EEG measurements and processing procedures.

**Additional file 6.** Cytokine assay with ELISA and RT-PCR.

**Additional file 7.** The number of missing data for study outcomes.

**Additional file 8 **Changes in the levels of hemoglobin (A), hematocrit (B), and red blood cell (C) during the study period of 1 year. Legends: According to the protocol, laboratory results were monitored at screening (V1), baseline (V2), 10 days (V5), 1-month (V7) and 1-year (V10) after EPO administration. Groups A and C administered with EPO showed higher levels of hemoglobin, hematocrit and red blood cell at 10 days and 1 month compared to groups B and D not treated with EPO (all *P* values < 0.001 by Kruskal-Wallis test). Bars represent SE. Abbreviations: EPO, erythropoietin; Hct, hematocrit; Hgb, hemoglobin; RBC, red blood cell; UCB, umbilical cord blood.

**Additional file 9.** The distribution of adverse events during study period of 12 months.

**Additional file 10.** Comparison of score changes in primary outcome measures.

**Additional file 11.** Comparison of score changes in ratio to baseline in primary outcome measures.

**Additional file 12.** Composition of allogeneic UCB units for groups A and B.

**Additional file 13.** Survey of parent perception of the intervention. Legends: Satisfaction towards the intervention was surveyed among the caregivers after the patients completed the trial before notified of the group assignment, and caregivers of 63 patients completed the survey (response rate of 71.6%). All items were positive numbers. In comparison among four groups, the items of language and mental function were significantly different (*P* = 0.05, 0.015 respectively). In satisfaction of caregivers in aspect of language function (A), the caregivers of group A agreed more strongly that the language abilities of their children had improved compared to group D (*P* = 0.05). For the satisfaction of caregivers in aspect of mental function (B), the caregivers of group B agreed more strongly that the cognitive ability improved compared to group D (*P* = 0.015).

**Additional file 14.**Subgroup comparisons of GMPM change ratios among 4 groups. Legends: Panel A shows subgroup analyses using GMPM change ratios according to (a) severe (GMFCS levels IV and V) vs. (b) mild (GMFCS levels I to III) impairment while Panel B shows GMPM change ratios according to (a) preterm (GA < 37 weeks) vs. (b) term (GA ≥ 37 weeks) birth. Among severely impaired subjects (*n* = 55, A: 14, B: 15, C: 10, D: 16), group A showed a larger improvement in the GMPM change ratio at 1 month and 12 months post-treatment than group D (*P* = 0.028 and *P* = 0.008, respectively) (Panel A-(a)). In term birth subgroup (*n* = 23, A: 6, B: 4, C: 4, D: 9), groups A and B showed significant improvement in the GMPM change ratio at 1, 6 month and 12 months post-treatment compared to groups C and D (*P* = 0.003, *P* = 0.029 and *P* = 0.011, respectively) (Panel B-(b)). Abbreviations: CP, cerebral palsy; EPO, erythropoietin; GA, gestational age; GMFCS, gross motor function classification system; UCB, umbilical cord blood.

**Additional file 15.** Changes in FA value in children aged over 3 years/ Legends: In the subgroup analysis classified by median ages of four groups - younger aged subgroups (aged below 3 years; *n* = 49, median age 2.28*y*; A: 12, B: 14, C: 10, D: 13) vs. older subgroup (aged over 3 years; *n* = 39, median age 4.12*y*; A: 10, B: 10, C: 10, D: 9). Primary outcome measures did not show any significant differences between four groups. FA change ratio in right anterior thalamic radiation (ATR) between baseline and 12 months after intervention are depicted in this figure. Group A showed significant difference of FA change ratio in ATRR compared with group D. *P < 0.05 by post-hoc analysis after Kruskal-Wallis test comparing the difference among 4 groups. Abbreviations: ATR: anterior thalamic radiation; FA, fraction anisotropy.

**Additional file 16.** Metabolic changes after UCB injection Legends: In comparison between pre- and post-intervention of PET/CT in each group, glucose metabolism of (A) bilateral cerebellar hemisphere increased in group B, (B) while the metabolic activity increased in midbrain and thalamus in group D. There were no meaningful changes in groups A and C. Data of 71 subjects were included in PET/CT analysis because, 7 did not undergo PET/CT on 12 months post-intervention, and 10 PET/CT imaging data were not appropriately processed due to anatomical distortion on SPM 12. Abbreviations: PET/CT, positron emission tomography/computed tomography; UCB, umbilical cord blood.

**Additional file 17.** Gene expression of IL-1β among responders vs. non-responders in groups A and B. Legends: Group A (UCB + EPO) and group B (UCB + P-EPO) were re-grouped into 2 groups as responder subgroup and non-responder subgroup, where responder (*n* = 13) subgroup refers to those who showed improvements in GMFCS levels and non-responders (*n* = 20) refers to those who did not show improvements in GMFCS levels at 12 months post-intervention. Gene expression assay with RT-PCR showed bigger increment in IL1-β mRNA level in their relative values to the baseline level (D-4) at 3 d (D+3; *P* = 0.032) and 10 d (D+10; *P* = 0.013) post-intervention when comparing responder subgroup (dark-pink) with non-responder subgroup (light pink). *P < 0.05 by Mann-Whitney U test. Abbreviations: GMFCS, Gross Motor Functional Classification System; IL, interleukin; RT-PCR, reverse transcription polymerase chain reaction.

**Additional file 18.**The *cytokine* analysis between responders and non-responders in group B.

## Data Availability

The datasets used and/or analyzed during the current study are available from the corresponding author on reasonable request.

## Abbreviations

AE: Adverse event
ATR: Anterior thalamic radiation
BSID-II: Bayley Scales for Infant Development-II
CP: Cerebral palsy
DAR: Delta/alpha band power ratio
EEG: Electroencephalography
EPO: Erythropoietin
FA: Fractional anisotropy
^18^F-PET/CT: ^18^F-fluorodeoxyglucose positron emission tomography/computed tomography
GMFCS: Gross motor function classification system
GMFM: Gross motor functional measure
GMPM: Gross motor performance measure
HLA: Human leukocyte antigen
IL: Interleukin
MRI: Magnetic resonance imaging
PTX3: Pentraxin 3
TNC: Total nucleated cell
UCB: Umbilical cord blood
