# Supplementary material for: Potentiation of cord blood cell therapy with erythropoietin for children with CP: a 2 × 2 factorial randomized placebo-controlled trial
Source: Stem Cell Res Ther. 2020 Nov 27;11:509. doi: 10.1186/s13287-020-02020-y (PMC7694426; doi:10.1186/s13287-020-02020-y)
Supplement: Supplementary file 1 — Additional file 1. Primary functional outcome measurements. [file 13287_2020_2020_MOESM1_ESM.pdf]

Additional file 1. Primary functional outcome measurements

Primary outcomes were changes in total scores of the Gross Motor Performance Measure (GMPM), Gross Motor Functional Measure (GMFM), and raw scores of Mental and Motor scales of the Bayley Scales for Infant Development-II (BSID-II).[1-3] The GMPM measures gross motor performance related to quality of movement and body controlling ability[4] and the GMFM measures gross motor function related to mobility[3]. The BSID-II was used to measure neurodevelopmental progress up to the ability of 42 month-old. The reliabilities of primary outcomes among our assessors were established in the clinical study team.[5-7]

1) Gross motor performance measure (GMPM)
   - Dissociated Movement, Coordination, Alignment, Weight shift, and Stability are rated. Each raw score (1-5 point) and converted percent scores are evaluated.
   - Range: 0-100
   - Higher value means better gross motor function.
2) Gross motor function measure (GMFM)
   - For 5 dimensions A through E, each raw score (0, 1, 2, 3, NT) and their summed score are converted into percent score, and score on each dimension is evaluated (A: Lying & Rolling, B: Sitting, C: Crawling and Kneeling, D: Standing, E: Walking, Running & Jumping).
   - Range: 0-100
   - Higher value means better gross motor function.
3) Bayley scales of infant development-II (BSID-II) – row score on motor and mental scale
   - The motor and mental function of children can be rated in terms of absolute status in unit of month and difference relative to reference by comparison each raw score and its reference score for age. In the study, changes of each raw score will be evaluated.
   - Range: 0-112 for motor scale and 0-178 for mental scale
   - Higher value means better gross motor function.

References to Supporting Information 1.

1. Boyce WF, Gowland C, Rosenbaum PL, Lane M, Plews N, Goldsmith CH, et al. The Gross Motor Performance Measure: validity and responsiveness of a measure of quality of movement. Phys Ther 1995;75:603-13.
2. Bayley N. Bayley Scales of Infant Development. Second Edition ed: The Psychological Corporation; 1993.
3. Dianne J Russell PLR, Lisa M Avery, Mary Lane. Gross Motor Function Measure (GMFM-66 & GMFM-88) User's Manual. London: Mac Keith Press; 2002.
4. Wright FV, Rosenbaum P, Fehlings D, Mesterman R, Breuer U, Kim M. The Quality Function Measure: reliability and discriminant validity of a new measure of quality of gross motor movement in ambulatory children with cerebral palsy. Dev Med Child Neurol 2014;56:770-8.
5. Ko J, Kim M. Inter-rater Reliability of the K-GMFM-88 and the GMPM for Children with Cerebral

Palsy. Ann Rehabil Med 2012;36:233-9.
6. Ko J, Kim M. Reliability and responsiveness of the gross motor function measure-88 in children with cerebral palsy. Phys Ther 2013;93:393-400.
7. Lee JH, Lim HK, Park E, Song J, Lee HS, Ko J, et al. Reliability and Applicability of the Bayley Scale of Infant Development-II for Children With Cerebral Palsy. Ann Rehabil Med 2013;37:167-74.
