# Supplementary material for: Potentiation of cord blood cell therapy with erythropoietin for children with CP: a 2 × 2 factorial randomized placebo-controlled trial
Source: Stem Cell Res Ther. 2020 Nov 27;11:509. doi: 10.1186/s13287-020-02020-y (PMC7694426; doi:10.1186/s13287-020-02020-y)
Supplement: Supplementary file 2 — Additional file 2. Secondary functional outcome measurements and survey of parent perception of the intervention. [file 13287_2020_2020_MOESM2_ESM.pdf]

Additional file 2. Secondary functional outcome measurements and survey of parent perception of the intervention

Secondary outcomes were Gross Motor Function Classification System (GMFCS),[1] Pediatric Evaluation of Disability Inventory,[2] Functional Independence Measure for Children,[3] summed scores on muscular strength by Medical Research Council scale,[4] Beery-Buktenica developmental test of visual-motor integration,[5] selective control assessment of lower extremity,[6] modified Ashworth scale,[7] modified Tardieu scale for hamstring[8] and Quality of Upper Extremity Skills Test.[9] Typical movements at each assessment were recorded using a digital camcorder with the consent. Also, the survey of parent perception of the intervention was performed among the caregivers.

1) Gross Motor Function Classification System (GMFCS)
   - Self-initiated movement is evaluated in terms of sitting, mobility, transfers.
   - Rating: Level 1, 2, 3, 4, and 5
   - Higher level means better gross motor function.
2) Pediatric Evaluation of Disability Inventory
   - Self-care, mobility, social function is rated. Changes of raw score are observed using raw score (0 = able, 1 = unable) and standard score per age group. In some cases, these values are converted to percent of each age group for statistical test.
   - Range: 0-100
   - Higher value means better function in categories of self-care, mobility, social function.
3) Functional Independence Measure for Children
   - This instrument contains 18 measurement items that are divided into 6 areas: self-care, sphincter control, transfers, locomotion, communication, and social cognition. A 7-level ordinal rating system ranging from 7 (complete independence) to 1 (total assistance) is used to rate performance.
   - Range: 18-126
   - Higher value means better independency.

Score	Rating	Score	Rating
1	Total Assistance	5	Supervision or Setup
2	Maximal Assistance	6	Modified Independence
3	Moderate Assistance	7	Complete Independence
4	Minimal Contact Assistance		

4) Muscular strength by Medical Research Council scale
   - This instrument is rated by Medical Research Council scales.
   - Rating: Zero, 0; Trace, 1; Poor, 2; Fair, 3; Good, 4; Normal, 5
   - The sum of MMT on both shoulder joints, flexors, extensors, abductors, and adductors of hip joints, both elbow joints, flexors and extensors of knee joints, and dorsiflexors and plantarflexors

of ankle joints

- Range: 0-160
- Higher value means stronger muscle.

5) Beery-Buktenica developmental test of visual-motor integration

- Visual perception test
- Raw score and percentage of Beery VMI, visual perception, motor coordination is utilized.
- Range: 0-30
- Higher value means better visuospatial function.

6) Selective control assessment of lower extremity

- Selective control abilities for hip, knee, ankle, subtalar joint, toes are rated.
- Scoring: Normal: 2, Impaired: 1, Unable: 0
- Higher value means better lower extremity function.

7) Modified Ashworth scale

- Biceps in upper extremity, hip adductors, hamstrings, and heel cords in lower extremity are measured bilaterally, and classified in to grades, which are evaluated according to the following criteria.

Grade 0	No increase in muscle tone
Grade 1	Slight increase in muscle tone, manifested by a catch and release or by minimal resistance at the end of the range of motion when the affected part(s) is moved in flexion or extension
Grade 1+	Slight increase in muscle tone, manifested by a catch, followed by minimal resistance throughout the remainder (less than half) of the ROM
Grade 2	More marked increase in muscle tone through most of the ROM, but affected part(s) easily moved
Grade 3	Considerable increase in muscle tone, passive movement difficult
Grade 4	Affected part(s) rigid in flexion or extension

8) Modified Tardieu scale

- A difference between R1 and R2 (R2-R1) is compared.
  R1 (the angle of catch following a fast velocity stretch)
  R2 (passive range of motion following a slow velocity stretch)
- A large difference between R1 and R2 suggests a large dynamic component in spasticity

9) Quality of upper extremity skills test

- Mobility of upper extremity is rated in terms of four domains including dissociated movement, grasp, protective extension, and weight bearing.
- Scoring
  Yes: able to complete item according to specification (scoring 2)
  No: cannot or will not complete item (scoring 1)

NT: not able to administer item (scoring 1)

- Range: 0-100 (for domain of grasp, score below 0 is possible)
- Higher value means better upper extremity function.

10) Survey of parent perception of the intervention

- Parent perception of the intervention was surveyed among the caregivers of the subjects.
- Scoring

+2	Strongly agree
+1	Agree
0	Undecided
-1	Disagree
-2	Strongly disagree

- Scores of each 9 items as below were compared among different groups.

		-2	-1	0	1	2
1	I am generally satisfied with the effect of cell therapy.					
2	The cell therapy process was not difficult and tolerable.					
3	There were no serious side effects during or after cell therapy.					
4	After cell therapy, motor ability such as muscle strength, sitting, and standing improved.					
5	After cell therapy, fine movement (painting, using chopsticks, etc.) improved.					
6	After cell therapy, cognitive ability (situation understanding, response to instructions, judgment, memory) improved.					
7	After cell therapy, language skill (understanding, pronunciation, and sentence expression) improved.					
8	After cell therapy, emotions or impulse control or sociality with others improved.					
9	After cell therapy, activities of daily living improved.					

References to Additional File 2.

1. Palisano R, Rosenbaum P, Walter S, Russell D, Wood E, Galuppi B. Development and reliability of a system to classify gross motor function in children with cerebral palsy. Dev Med Child Neurol 1997;39:214-23.
2. Stephen M. Haley WJC, Larry H. Ludlow, Jane T. Haltiwanger, Peter J. Andrellos. Pediatric Evaluation of Disability Inventory (PEDI). Boston, MA: PEDI Research Group; 1992.
3. Uniform Data System for Medical Rehabilitation. Guide for the Uniform Data Set for Medical Rehabilitation for Children (WeeFIM). State University of New York at Buffalo; 1993.
4. Paternostro-Sluga T, Grim-Stieger M, Posch M, Schuhfried O, Vacariu G, Mittermaier C, et al. Reliability and validity of the Medical Research Council (MRC) scale and a modified scale for testing muscle strength in patients with radial palsy. J Rehabil Med 2008;40:665-71.
5. Beery KE, Buktenica NA, Beery NA. The Beery-Buktenica Developmental Test of Visual-Motor

Integration : for children and adults (Beery VMI). Parsippany, N.J.: Pearson; 2010.

6. Fowler EG, Staudt LA, Greenberg MB, Oppenheim WL. Selective Control Assessment of the Lower Extremity (SCALE): development, validation, and interrater reliability of a clinical tool for patients with cerebral palsy. Dev Med Child Neurol 2009;51:607-14.

7. Yam WK, Leung MS. Interrater reliability of Modified Ashworth Scale and Modified Tardieu Scale in children with spastic cerebral palsy. J Child Neurol 2006;21:1031-5.

8. Boyd RN, Graham HK. Objective measurement of clinical findings in the use of botulinum toxin type A for the management of children with cerebral palsy. European Journal of Neurology 1999;6:s23-s35.

9. DeMatteo C LM, Russell D, Pollock N, Rosenbaum P, Walter S. QUEST: Quality of Upper Extremity skills Test Manual. Hamilton, Ontario: Neurodevelopmental Research Unit, Chedoke Campus, Chedoke-McMasters Hospital; 1992.
