# Supplementary material for: Potentiation of cord blood cell therapy with erythropoietin for children with CP: a 2 × 2 factorial randomized placebo-controlled trial
Source: Stem Cell Res Ther. 2020 Nov 27;11:509. doi: 10.1186/s13287-020-02020-y (PMC7694426; doi:10.1186/s13287-020-02020-y)
Supplement: Supplementary file 3 — Additional file 3. Brain MRI measurements and processing procedures. [file 13287_2020_2020_MOESM3_ESM.pdf]

Additional file 3. Brain MRI measurements and processing procedures

All participants underwent brain MRI using a 3T GE Signa System (General Electric, Milwaukee, WI, USA). Diffusion tensor image (DTI) data were acquired using 2D axial spin echo echo planar imaging with refocusing pulses. The sequence parameters were TR/TE of 12,000/108 ms; 1 NEX, 66 slices; 22-cm FOV; 128 ´128 matrix; 2.0 mm slice thickness; 25 gradient directions; *B* = 1000; and a non-diffusion-weighted baseline image (*B* = 0).

The imaging data were then processed using FSL software (created by the Analysis Group, FMRIB, Oxford, UK). Fractional anisotropy (FA) values were calculated by voxel-based approach using TBSS (Tract-Based Spatial Statistics) tool in an automated process. A total of 17 different white-mater tracts from JHU white-matter tractography atlases were analyzed: single corpus callosum and bilateral fibers of eight tracts such as anterior thalamic radiation (ATR), cingulum in the cingulate cortex area, cingulum in the hippocampal area, corticospinal tract, inferior fronto-occipital fasciculus, superior and inferior longitudinal fasciculus, and uncinate fasciculus. Registration of MRI data to the templates was made using FMRIB's linear image registration tool (FLIRT). The changes in FA from baseline to the 12 months post-intervention were used to determine the effects of the treatment on white matter integration. The ratios of FA variances between pre-intervention and 12-month post intervention were calculated: ((*FA values of post-intervention – FA values of pre-intervention) / FA values of pre-intervention*).
