# Supplementary material for: Potentiation of cord blood cell therapy with erythropoietin for children with CP: a 2 × 2 factorial randomized placebo-controlled trial
Source: Stem Cell Res Ther. 2020 Nov 27;11:509. doi: 10.1186/s13287-020-02020-y (PMC7694426; doi:10.1186/s13287-020-02020-y)
Supplement: Supplementary file 4 — Additional file 4. PET/CT measurements and processing procedures. [file 13287_2020_2020_MOESM4_ESM.pdf]

Additional file 4. PET/CT measurements and processing procedures

18F-Fluorodeoxyglucose(18F-FDG) positron emission tomography/computed tomography (PET/CT) images were acquired using a PET/CT scanner (Biograph mCT, Siemens, Germany). Before radiotracer injection, a transmission scan was performed using CT to generate the attenuation maps. Approximately 50 minutes after the intravenous administration of 74 MBq of 18F-FDG and 30 minutes after sleep induction with chloral hydrate, 90 slices of brain emission images were obtained over a period of 20 minutes. The data from the PET images were analyzed by image reconstruction using the row action maximum likelihood algorithm. Two board-certified nuclear physicians reviewed the 18F-FDG-PET/CT scans. The spatial, preprocessing, and statistical analyses were performed using SPM12 (Institute of Neurology, University College of London, UK) implanted in Matlab R2018a (Mathworks, MA, USA) to assess differences in the regional brain glucose metabolism between the groups and between the pre-treatment and post-treatment image data. The 18F-FDG-PET images were converted from DICOM to the ANALYZE format (.nii) using DICOM Import on SPM12. The data were then normalized to a standard PET template provided by SPM12. The standardized data were then smoothed using a Gaussian kernel (full-width half-maximum, 16 mm). Variables were compared by Student’s t-tests for two paired groups. For comparison of four groups, ANOVA with Tukey post-hoc tests were used. The changes in the images from pre-treatment to post-treatment in each group were compared using paired *t*-tests. Voxels with an uncorrected $P < 0.05$ were considered significant, and the extent threshold *K*e was set at 100 voxels.
