# Supplementary material for: Potentiation of cord blood cell therapy with erythropoietin for children with CP: a 2 × 2 factorial randomized placebo-controlled trial
Source: Stem Cell Res Ther. 2020 Nov 27;11:509. doi: 10.1186/s13287-020-02020-y (PMC7694426; doi:10.1186/s13287-020-02020-y)
Supplement: Supplementary file 5 — Additional file 5. EEG measurements and processing procedures. [file 13287_2020_2020_MOESM5_ESM.pdf]

Additional file 5. EEG measurements and processing procedures

Asleep electroencephalogram (EEG) was taken using 19 channel EEG machine (Comet-PLUS® Grass technologies, U.S.A.), and the waves were converted to XX file and was analyzed by using a special software program (iSyncBrain(@) version 2.0, iMediSync Inc., Korea). Average delta (1-4Hz), theta (4-8Hz), alpha (8-12Hz), beta (12-20Hz), and gamma (30-45Hz) band were computed and the topomaps of relative band powers, which was calculated by total absolute power per 1 Hz frequency, were drawn on 2D brain image. Average delta/alpha band power ratio (DAR) was obtained from five regions of brain which were frontal, central, temporal, parietal, and occipital cortices, and their differences from pre-treatment to 12 months post-treatment were also depicted. Mann-Whitney U test was used to compare the difference between pre- and post-treatment DAR from each brain regions. For comparison of four groups, ANOVA with Tukey post-hoc tests were used.
