# Supplementary material for: Potentiation of cord blood cell therapy with erythropoietin for children with CP: a 2 × 2 factorial randomized placebo-controlled trial
Source: Stem Cell Res Ther. 2020 Nov 27;11:509. doi: 10.1186/s13287-020-02020-y (PMC7694426; doi:10.1186/s13287-020-02020-y)
Supplement: Supplementary file 6 — Additional file 6. Cytokine assay with ELISA and RT-PCR. [file 13287_2020_2020_MOESM6_ESM.pdf]

Additional file 6. Cytokine assay with ELISA and RT-PCR

Blood samples were collected in EDTA Vacutainer® tube at 4 days before UCB injection (D-4) and just before cyclosporine administration, at the day of UCB injection prior to injection (D-0) , and 3, 10, and 30 days after UCB injection (D+3, D+10, and D+30), respectively. The blood samples were separated to plasma and buffy coats by centrifugation at 2,000 *g* for 20 minutes in room temperature, and then were preserved at -80℃ in a freezer for assay. Plasma levels of PTX3, interleukin (IL)-8, TNF-α, and IL-1β were measured using enzyme-linked immunosorbent assay (ELISA) test kits (R&D Systems, Minneapolis, USA), using the same method as in our previous study.[1] After diluting the EDTA plasma samples with reagent diluent at 23% (*v/v*) for PTX3 and 50% (*v/v*) for IL-8, biotinylated antibody followed by horseradish peroxidase-conjugated anti-streptovidin antibody were tagged to detect the targeted proteins. Then, marked proteins were visualized after addition of 100 μL of 3,3',5,5'-tetramethylbenzidine for 30 minutes, followed by 50 μL of the stop solution. Every steps of procedure were repeated and each mean value was used for analyses.

Reverse transcription polymerase chain reaction (RT-PCR) was also done by using ReverTra Ace® qPCR RT Master Mix (Toyobo Co., Osaka, Japan) for cDNA synthesis. All reactions were run in an Applied Biosystems Veriti 96 Well Thermal Cycler, with a 3 min hot start at 95℃, followed by 37 cycles of a three-step thermocycling program: denaturation, 30 s at 95℃; annealing: 30 s at 63℃ (18s RNA for 57℃), and extension: 30 s at 72℃. After three times conduction of RT-PCR, each mean value was used for analyses.

	Primer sequences (5'-3;)		
Genes	Forward	Reverse	Size (bp)
PTX3	AATGCATCTCCTTGCGATTC	TGAAGTGCTTGTCCCATTCC	182
TNF-α	GAGTGACAAGCCTGTAGCCCATGTTGTAGCA	GCAATGATCCCAAAGTAGACCTGCCCAGACT	444
IL-1β	AAACAGATGAAGTGCTCCTTCCAGG	TGGAGAACACCACTTGTTGCTCCA	391
IL-8	ATGACTTCCAAGCTGGCCGTGGCT	TCTCAGCCCTCTTCAAAAACTTCTC	292
18s rRNA	CCGCAGCTAGGAATAATGGA	CCCTCTTAATCATGGCCTCA	76

References to Supporting Information 6.

1. Kang M, Min K, Jang J, Kim SC, Kang MS, Jang SJ, et al. Involvement of Immune Responses in the Efficacy of Cord Blood Cell Therapy for Cerebral Palsy. Stem Cells Dev 2015;24:2259-68.
