# Supplementary material for: Potentiation of cord blood cell therapy with erythropoietin for children with CP: a 2 × 2 factorial randomized placebo-controlled trial
Source: Stem Cell Res Ther. 2020 Nov 27;11:509. doi: 10.1186/s13287-020-02020-y (PMC7694426; doi:10.1186/s13287-020-02020-y)
Supplement: Supplementary file 7 — Additional file 7. The number of missing data for study outcomes. [file 13287_2020_2020_MOESM7_ESM.pdf]

Additional file 7. The number of missing data for study outcomes

	Assessment period[a]	Group[b] (*n* = 88)			
		Group A (*n* = 22)	Group B (*n* = 24)	Group C (*n* = 20)	Group D (*n* = 22)
**GMFM**	Baseline	0	0	0	0
	1 month	0	0	0	1
	3 months	0	0	0	0
	6 months	0	0	0	0
	12 months	1	2	0	2
**GMPM**	Baseline	0	0	0	0
	1 month	0	0	0	1
	3 months	0	0	0	0
	6 months	0	0	0	0
	12 months	1	2	0	2
**BSID-II Mental scale *raw score***	Baseline	0	0	0	0
	1 month	0	0	0	1
	3 months	0	0	0	0
	6 months	0	0	0	0
	12 months	1	2	0	2
**BSID-II Motor scale *raw score***	Baseline	0	0	0	0
	1 month	0	0	0	1
	3 months	0	0	0	0
	6 months	0	0	0	0
	12 months	1	2	0	2
**DTI**	Baseline	0	0	1[c]	0
	12 months	2[c]	3[c]	1[c]	2[c]
**PET/CT**	Baseline	0	0	0	2
	12 months	2	3+(5)[c]	0	2+(5)[c]
**EEG**	Baseline	0	2	0	0
	12 months	2	2+(2)[c]	0	2+(1)[c]
**Cytokine ELISA[d]**	D-4	7	6	3	5
	D-0	7	6	3	5
	D+3	7	6	4	5
	D+10	7	6	3	5
	D+30	7	6	6	5
**Cytokine RT-PCR[d]**	D-4	7	6	3	5
	D-0	7	6	3	5
	D+3	7	6	3	5
	D+10	7	6	4	5
	D+30	7	6	6	5

[a]Assessment periods are each months after intervention. [b]Group A (n = 22) received UCB and EPO, group B (n = 24) received UCB and placebo EPO, group C (n = 20) received placebo UCB and EPO, and group D (n = 22) received placebo UCB and placebo EPO. [c]numbers are the missing data who refused or failed to perform the evaluation, and the numbers in the parenthesis are ones that could not be appropriately processed through the software programs. [d]Cytokine analyses were performed from blood samples collected at 4 days before UCB injection (D-4), at the day of and prior to UCB injection (D-0), and 3, 10, and 30 days after UCB injection (D+3, D+10, and D+30, respectively).

Abbreviations: BSID-II, Korean version of the Bayley scales of infant development, second edition; DTI, diffusion tensor image; GMPM, gross motor performance measure; GMFM, gross motor function measure; PET/CT, positron emission tomographic/computed tomography; EEG, electroencephalogram; ELISA, enzyme linked immunosorbent assay; RT-PCR, reverse transcription polymerase chain reaction
