# Supplementary figures and images for: Potentiation of cord blood cell therapy with erythropoietin for children with CP: a 2 × 2 factorial randomized placebo-controlled trial
Source: Stem Cell Res Ther. 2020 Nov 27;11:509. doi: 10.1186/s13287-020-02020-y (PMC7694426; doi:10.1186/s13287-020-02020-y)

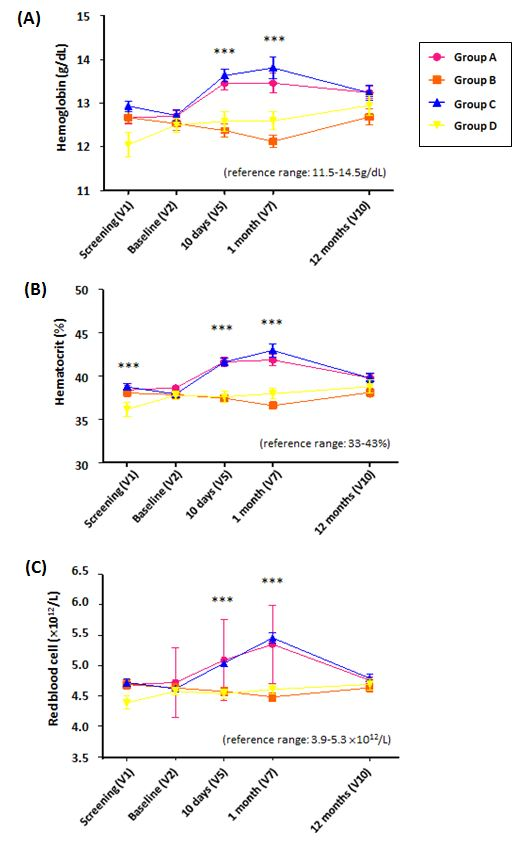

Supplement: Supplementary file 8 — Additional file 8 Changes in the levels of hemoglobin (A), hematocrit (B), and red blood cell (C) during the study period of 1 year. Legends: According to the protocol, laboratory results were monitored at screening (V1), baseline (V2), 10 days (V5), 1-month (V7) and 1-year (V10) after EPO administration. Groups A and C administered with EPO showed higher levels of hemoglobin, hematocrit and red blood cell at 10 days and 1 month compared to groups B and D not treated with EPO (all P values < 0.001 by Kruskal-Wallis test). Bars represent SE. Abbreviations: EPO, erythropoietin; Hct, hematocrit; Hgb, hemoglobin; RBC, red blood cell; UCB, umbilical cord blood. [file 13287_2020_2020_MOESM8_ESM.tif]

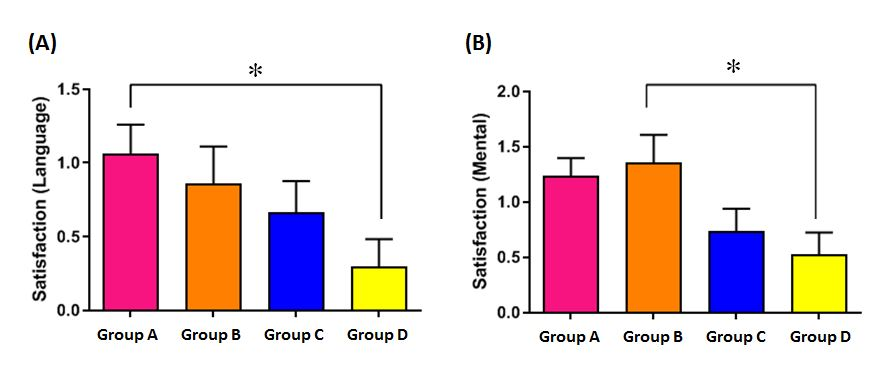

Supplement: Supplementary file 13 — Additional file 13. Survey of parent perception of the intervention. Legends: Satisfaction towards the intervention was surveyed among the caregivers after the patients completed the trial before notified of the group assignment, and caregivers of 63 patients completed the survey (response rate of 71.6%). All items were positive numbers. In comparison among four groups, the items of language and mental function were significantly different (P = 0.05, 0.015 respectively). In satisfaction of caregivers in aspect of language function (A), the caregivers of group A agreed more strongly that the language abilities of their children had improved compared to group D (P = 0.05). For the satisfaction of caregivers in aspect of mental function (B), the caregivers of group B agreed more strongly that the cognitive ability improved compared to group D (P = 0.015). [file 13287_2020_2020_MOESM13_ESM.tif]

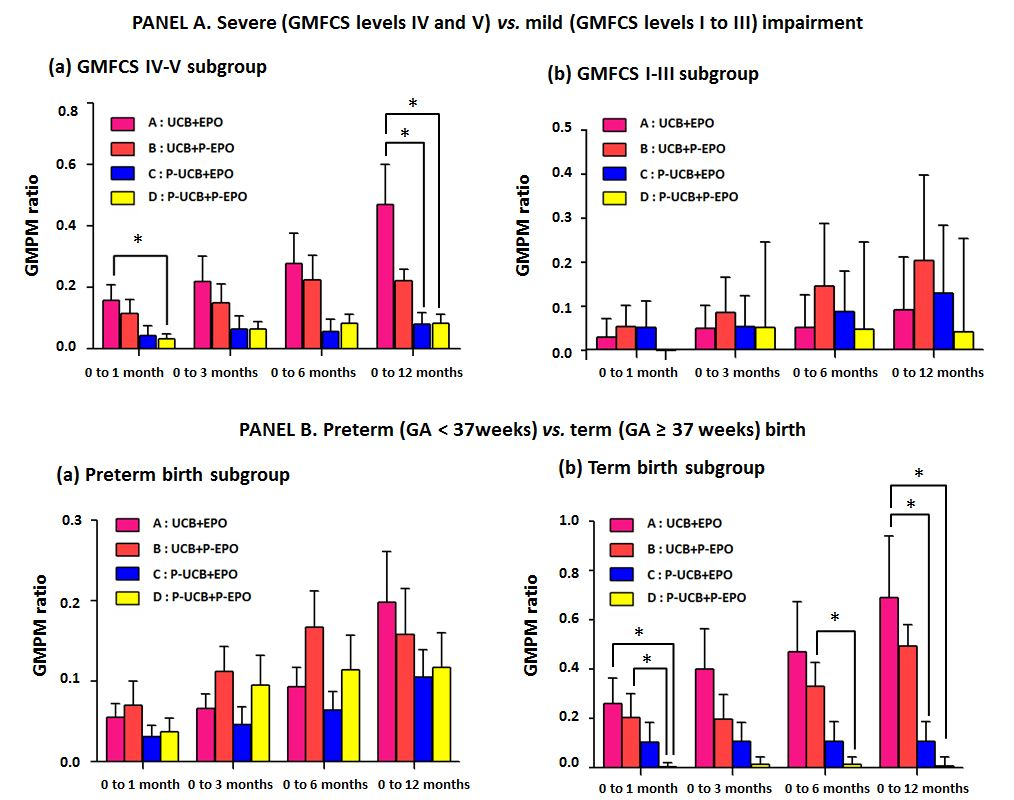

Supplement: Supplementary file 14 — Additional file 14.Subgroup comparisons of GMPM change ratios among 4 groups. Legends: Panel A shows subgroup analyses using GMPM change ratios according to (a) severe (GMFCS levels IV and V) vs. (b) mild (GMFCS levels I to III) impairment while Panel B shows GMPM change ratios according to (a) preterm (GA < 37 weeks) vs. (b) term (GA ≥ 37 weeks) birth. Among severely impaired subjects (n = 55, A: 14, B: 15, C: 10, D: 16), group A showed a larger improvement in the GMPM change ratio at 1 month and 12 months post-treatment than group D (P = 0.028 and P = 0.008, respectively) (Panel A-(a)). In term birth subgroup (n = 23, A: 6, B: 4, C: 4, D: 9), groups A and B showed significant improvement in the GMPM change ratio at 1, 6 month and 12 months post-treatment compared to groups C and D (P = 0.003, P = 0.029 and P = 0.011, respectively) (Panel B-(b)). Abbreviations: CP, cerebral palsy; EPO, erythropoietin; GA, gestational age; GMFCS, gross motor function classification system; UCB, umbilical cord blood. [file 13287_2020_2020_MOESM14_ESM.tif]

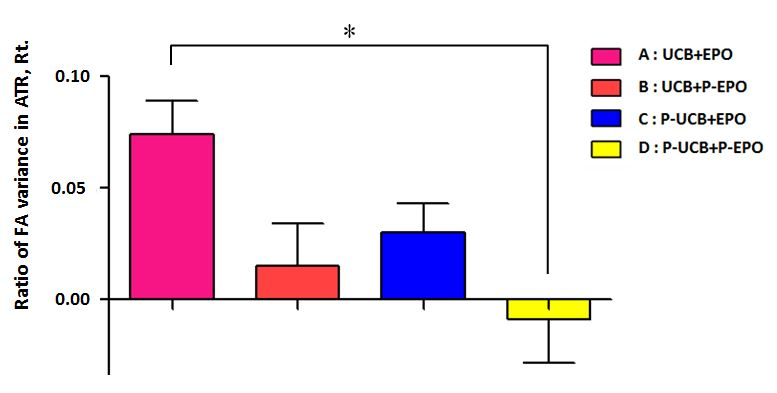

Supplement: Supplementary file 15 — Additional file 15. Changes in FA value in children aged over 3 years/ Legends: In the subgroup analysis classified by median ages of four groups - younger aged subgroups (aged below 3 years; n = 49, median age 2.28y; A: 12, B: 14, C: 10, D: 13) vs. older subgroup (aged over 3 years; n = 39, median age 4.12y; A: 10, B: 10, C: 10, D: 9). Primary outcome measures did not show any significant differences between four groups. FA change ratio in right anterior thalamic radiation (ATR) between baseline and 12 months after intervention are depicted in this figure. Group A showed significant difference of FA change ratio in ATRR compared with group D. *P < 0.05 by post-hoc analysis after Kruskal-Wallis test comparing the difference among 4 groups. Abbreviations: ATR: anterior thalamic radiation; FA, fraction anisotropy. [file 13287_2020_2020_MOESM15_ESM.tif]

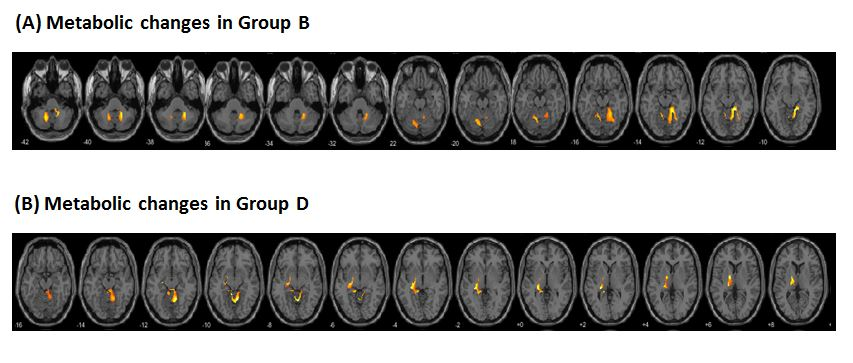

Supplement: Supplementary file 16 — Additional file 16. Metabolic changes after UCB injection Legends: In comparison between pre- and post-intervention of PET/CT in each group, glucose metabolism of (A) bilateral cerebellar hemisphere increased in group B, (B) while the metabolic activity increased in midbrain and thalamus in group D. There were no meaningful changes in groups A and C. Data of 71 subjects were included in PET/CT analysis because, 7 did not undergo PET/CT on 12 months post-intervention, and 10 PET/CT imaging data were not appropriately processed due to anatomical distortion on SPM 12. Abbreviations: PET/CT, positron emission tomography/computed tomography; UCB, umbilical cord blood. [file 13287_2020_2020_MOESM16_ESM.tif]

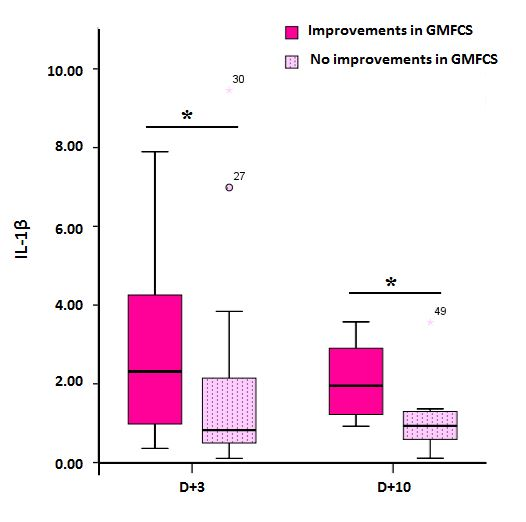

Supplement: Supplementary file 17 — Additional file 17. Gene expression of IL-1β among responders vs. non-responders in groups A and B. Legends: Group A (UCB + EPO) and group B (UCB + P-EPO) were re-grouped into 2 groups as responder subgroup and non-responder subgroup, where responder (n = 13) subgroup refers to those who showed improvements in GMFCS levels and non-responders (n = 20) refers to those who did not show improvements in GMFCS levels at 12 months post-intervention. Gene expression assay with RT-PCR showed bigger increment in IL1-β mRNA level in their relative values to the baseline level (D-4) at 3 d (D+3; P = 0.032) and 10 d (D+10; P = 0.013) post-intervention when comparing responder subgroup (dark-pink) with non-responder subgroup (light pink). *P < 0.05 by Mann-Whitney U test. Abbreviations: GMFCS, Gross Motor Functional Classification System; IL, interleukin; RT-PCR, reverse transcription polymerase chain reaction. [file 13287_2020_2020_MOESM17_ESM.tif]
