# Supplementary material for: Potentiation of cord blood cell therapy with erythropoietin for children with CP: a 2 × 2 factorial randomized placebo-controlled trial
Source: Stem Cell Res Ther. 2020 Nov 27;11:509. doi: 10.1186/s13287-020-02020-y (PMC7694426; doi:10.1186/s13287-020-02020-y)
Supplement: Supplementary file 9 — Additional file 9. The distribution of adverse events during study period of 12 months. [file 13287_2020_2020_MOESM9_ESM.pdf]

Additional file 9. The distribution of adverse events during study period of 12 months

	Group A[a] (n = 22)	Group B[a] (n = 24)	Group C[a] (n = 20)	Group D[a] (n = 22)	P-value[b]
**Serious adverse events[c]**					
Pneumonia	1[d]			1[d]	0.724
Seizure	1[d]			2 (1[d],1[e])	0.138
Otitis media acute	1[d]				0.727
Pyrexia		1[d]			1
Entropion			1[e]		0.227
Hepatitis viral			1[f]		0.227
Nasopharyngitis				1[f]	0.727
Labial frenectomy			1[d]		0.227
**Other adverse events**					
Upper respiratory infection	13	16	10	17	0.305
Pharyngitis	4	3	4		0.132
Rhinorrhea	1	1	1		0.894
Influenza		1			1
Sputum increased				1	0.727
Hand-foot mouth disease	1		1		0.472
Pyrexia	3	2	3		0.287
Diarrhoea			1		0.227
Ileus			1		0.227
Gastroenteritis				1	0.727
Pneumonia	1		4	1	0.058
Nausea	1		1	1	0.702
Decreased appetite				1	0.73
Constipation	5	3	7	6	0.353
Urticaria	2				0.17
Herpes infection		1			1
Seizure				2	0.17
Electroencephalogram abnormal		1		1	1
Cough	1				0.727
Abdomen pain		1			1
Chest pain		1			1
Bacteriuria			1		0.227
Hypertrichosis			1		0.227
Blood electrolyte increased			1		0.227
Hypoaldosteronism			1		0.227
Mucocutaneous rash	4	2	2		0.217
Skin abrasion		1			1
Dermatitis diaper				1	0.727
Eczema	1		1		0.472
Pruritus		2			0.242
Cellulitis		1		1	1
Dehydration			1	1	0.472

Tachycardia	2				0.17
Tachypnoea	1				0.727
Fatigue	1				0.727
Otitis media acute		1		1	1
Swelling of eyelid	1				0.727
Hordeolum		1			1

[a]Group A (n = 22) received UCB and EPO, group B (n = 24) received UCB and placebo EPO, group C (n = 20) received placebo UCB and EPO, and group D (n = 22) received placebo UCB and placebo EPO. [b]P-values were calculated for difference among four groups of the number of patients with reported adverse events using Fisher's exact analysis. [c]Serious adverse events were defined as any event, resulting in death, life-threatening, requiring hospitalization or prolongation of hospital stay. Relationship with the intervenition in serious adverse events were shown as [d]unlikely, [e]non-related or [f]cannot be evaluated.

The source of terminology was Medical Dictionary for Regulatory Activities (MedDRA) 21.1.
