# Supplementary material for: Potentiation of cord blood cell therapy with erythropoietin for children with CP: a 2 × 2 factorial randomized placebo-controlled trial
Source: Stem Cell Res Ther. 2020 Nov 27;11:509. doi: 10.1186/s13287-020-02020-y (PMC7694426; doi:10.1186/s13287-020-02020-y)
Supplement: Supplementary file 10 — Additional file 10. Comparison of score changes in primary outcome measures. [file 13287_2020_2020_MOESM10_ESM.pdf]

Additional file 10. Comparison of score changes in primary outcome measures

	Assessment interval	Group (*n* = 88)[a]				P-value[b]
		Group A (*n* = 22)	Group B (*n* = 24)	Group C (*n* = 20)	Group D (*n* = 22)	
**GMPM**	0-1 month	2.30 (0.47)	2.51 (0.69)	1.37 (0.53)	0.71 (0.42)	0.03[c]
	0-3 month	3.11 (0.59)	3.42 (0.72)	1.80 (0.69)	1.93 (1.06)	0.22
	0-6 month	3.90 (0.79)	4.93 (0.93)	2.43 (0.80)	2.42 (1.17)	0.14
	0-12 month	6.85 (1.30)	5.58 (1.31)	3.67 (1.08)	2.34 (1.23)	0.02[c]
**GMFM**	0-1 month	3.14 (0.47)	2.17 (0.44)	4.30 (0.86)	2.28 (0.56)	0.07
	0-3 month	5.23 (0.70)	4.25 (0.68)	6.85 (1.21)	5.05 (1.01)	0.21
	0-6 month	6.50 (0.71)	6.92 (1.10)	10.20 (1.50)	6.86 (1.15)	0.16
	0-12 month	8.86 (1.08)	9.33 (1.62)	13.30 (1.83)	8.27 (1.29)	0.15
**BSID-II Mental scale** ***raw score***	0-1 month	4.59 (1.08)	5.00 (1.38)	5.35 (0.95)	3.77 (1.07)	0.30
	0-3 month	7.55 (1.23)	9.33 (1.88)	8.90 (1.35)	8.14 (1.38)	0.93
	0-6 month	12.86 (1.83)	13.38 (2.16)	16.00 (2.58)	10.50 (1.64)	0.49
	0-12 month	21.18 (3.48)	19.91 (2.71)	23.95 (4.03)	15.05 (2.43)	0.39
**BSID-II Motor scale** ***raw score***	0-1 month	2.23 (1.13)	1.71 (0.45)	3.95 (1.36)	2.82 (1.01)	0.49
	0-3 month	5.14 (1.84)	4.33 (0.89)	5.80 (1.76)	4.50 (1.18)	0.91
	0-6 month	4.91 (1.55)	5.96 (1.01)	9.05 (2.01)	3.36 (2.84)	0.31
	0-12 month	8.45 (1.86)	9.05 (2.01)	12.40 (2.36)	7.18 (1.43)	0.46

Values are *(scores at the time point) – (scores at baseline)*, and shown as mean (SE). [a]Group A (n = 22) received UCB and EPO, group B (n = 24) received UCB and placebo EPO, group C (n = 20) received placebo UCB and EPO, and group D (n = 22) received placebo UCB and placebo EPO. [b]P-values were calculated for difference among four groups of the score changes in four main functional assessments using Kruskal-Wallis test. [c]P < .05, when assessed by post hoc analysis after Kruskal Wallis test on GMPM ratio at 0-1 month and 0-12 month, *significant difference were shown* among group 1 *vs*. group 4.

Abbreviations: BSID-II, Korean version of the Bayley scales of infant development second edition; GMPM, gross motor performance measure; GMFM, gross motor function measure.
