# Supplementary material for: Potentiation of cord blood cell therapy with erythropoietin for children with CP: a 2 × 2 factorial randomized placebo-controlled trial
Source: Stem Cell Res Ther. 2020 Nov 27;11:509. doi: 10.1186/s13287-020-02020-y (PMC7694426; doi:10.1186/s13287-020-02020-y)
Supplement: Supplementary file 11 — Additional file 11. Comparison of score changes in ratio to baseline in primary outcome measures. [file 13287_2020_2020_MOESM11_ESM.pdf]

Additional file 11. Comparison of score changes in ratio to baseline in primary outcome measures

		Group[a] (*n* = 88)				
	Assessment interval	Group A (*n* = 22)	Group B (*n* = 24)	Group C (*n* = 20)	Group D (*n* = 22)	P-value[b]
**GMPM**	0-1 month	0.11 (0.04)	0.09 (0.03)	0.05 (0.02)	0.02 (0.01)	0.02[c]
	0-3 month	0.16 (0.05)	0.13 (0.03)	0.06 (0.02)	0.06 (0.03)	0.14
	0-6 month	0.20 (0.07)	0.19 (0.04)	0.07 (0.02)	0.07 (0.03)	0.09
	0-12 month	0.33 (0.09)	0.21 (0.06)	0.11 (0.03)	0.07 (0.03)	0.02[c]
**GMFM**	0-1 month	0.14 (0.05)	0.10 (0.02)	0.12 (0.02)	0.10 (0.02)	0.69
	0-3 month	0.27 (0.12)	0.18 (0.03)	0.18 (0.03)	0.17 (0.03)	0.85
	0-6 month	0.32 (0.12)	0.31 (0.06)	0.28 (0.05)	0.25 (0.04)	0.67
	0-12 month	0.42 (0.16)	0.38 (0.07)	0.34 (0.05)	0.29 (0.06)	0.47
**BSID-II Mental scale *raw score***	0-1 month	0.05 (0.01)	0.05 (0.02)	0.05 (0.01)	0.04 (0.01)	0.43
	0-3 month	0.09 (0.02)	0.10 (0.02)	0.08 (0.02)	0.09 (0.02)	0.96
	0-6 month	0.14 (0.02)	0.15 (0.03)	0.15 (0.03)	0.11 (0.02)	0.95
	0-12 month	0.22 (0.04)	0.22 (0.03)	0.24 (0.05)	0.16 (0.03)	0.55
**BSID-II Motor scale *raw score***	0-1 month	0.04 (0.02)	0.04 (0.01)	0.06 (0.02)	0.15 (0.09)	0.66
	0-3 month	0.09 (0.03)	0.09 (0.02)	0.09 (0.03)	0.24 (0.10)	0.87
	0-6 month	0.09 (0.02)	0.14 (0.03)	0.15 (0.03)	0.24 (0.12)	0.66
	0-12 month	0.17 (0.03)	0.20 (0.04)	0.21 (0.04)	0.31 (0.11)	0.87

[a]Group A (n = 22) received UCB and EPO, group B (n = 24) received UCB and placebo EPO, group C (n = 20) received placebo UCB and EPO, and group D (n = 22) received placebo UCB and placebo EPO. [b]P-values were calculated for difference among four groups of the score changes in four main functional assessments using Kruskal-Wallis Test. [c]P < .05, when assessed by post hoc analysis after Kruskal-Wallis test on GMPM change ratio at 0-1 month and 0-12 month, *significant difference were shown* among group 1 *vs*. group 4.

Abbreviations: BSID-II, Korean version of the Bayley scales of infant development, second edition; GMPM, gross motor performance measure; GMFM, gross motor function measure.
