# Supplementary material for: Potentiation of cord blood cell therapy with erythropoietin for children with CP: a 2 × 2 factorial randomized placebo-controlled trial
Source: Stem Cell Res Ther. 2020 Nov 27;11:509. doi: 10.1186/s13287-020-02020-y (PMC7694426; doi:10.1186/s13287-020-02020-y)
Supplement: Supplementary file 12 — Additional file 12. Composition of allogeneic UCB units for groups A and B. [file 13287_2020_2020_MOESM12_ESM.pdf]

Additional file 12. Composition of allogeneic UCB units for groups A and B

Participant	Number of TNC (×10^7) per body weight (kg)	Viability (%)	CD34 (+) cells (%)*	Number of HLA mismatch†
**A1**	3.49	95	0.14	2
**A2**	4.97	97	0.13	2
**A3**	4.74	95	0.10	1
**A4**	4.00	98	1.12	2
**A5**	4.92	85	0.17	0
**A6**	7.32	93	0.16	2
**A7**	5.86	75	0.18	2
**A8**	3.03	92	0.25	2
**A9**	5.43	89	0.09	2
**A10**	4.86	91	0.23	1
**A11**	3.48	93	0.28	1
**A12**	5.12	90	0.25	1
**A13**	5.30	84	0.19	1
**A14**	4.25	95	0.18	1
**A15**	4.68	100	0.18	2
**A16**	5.97	96	0.11	1
**A17**	5.39	89	0.20	2
**A18**	5.66	91	0.30	2
**A19**	4.23	83	0.17	1
**A20**	3.60	95	0.44	2
**A21**	4.49	97	0.09	2
**A22**	3.58	85	0.28	1
**B1**	5.89	93	0.16	1
**B2**	4.82	93	0.11	0
**B3**	4.07	97	1.26	1
**B4**	5.44	94	0.16	1
**B5**	3.98	97	0.30	1
**B6**	3.19	94	0.12	2
**B7**	6.54	92	0.28	1
**B8**	8.67	100	0.72	0
**B9**	6.00	91	0.15	1
**B10**	4.82	96	0.24	2
**B11**	4.39	96	0.33	1
**B12**	5.23	84	0.62	2
**B13**	7.47	94	0.52	2
**B14**	3.59	99	0.27	1
**B15**	4.03	99	0.20	2
**B16**	4.14	90	0.67	2
**B17**	5.61	84	0.20	2
**B18**	4.89	80	0.44	2
**B19**	4.64	80	0.16	1
**B20**	5.24	80	0.41	0
**B21**	4.30	65	0.25	2
**B22**	7.62	76	0.11	2
**B23**	5.19	91	0.29	2

**B24**	9.27	87	0.61	1

A1-22 are participants included in Group A (UCB + EPO), and B1-24 are participants included in Group B (UCB + placebo EPO). *CD34 (+) cells (%) was calculated from dividing CD34 (+) cells by TNC in each unit. †UCB unit matched for four of six HLA types A, B, and DRB1 antigens at least with high resolution. 0, 1 and 2 represent full-matched (n=4), one mismatched (n = 19) and two mismatched (n = 23) antigens from six HLA antigens, respectively. Abreviations: TNC, total nucleated cells.
