# Supplementary material for: Potentiation of cord blood cell therapy with erythropoietin for children with CP: a 2 × 2 factorial randomized placebo-controlled trial
Source: Stem Cell Res Ther. 2020 Nov 27;11:509. doi: 10.1186/s13287-020-02020-y (PMC7694426; doi:10.1186/s13287-020-02020-y)
Supplement: Supplementary file 18 — Additional file 18.The cytokine analysis between responders and non-responders in group B. [file 13287_2020_2020_MOESM18_ESM.pdf]

Additional file 18. The cytokine analysis between responders and non-responders in group B

Ratio of PTX3 and IL-8 changes		Responders[a] (n=6)	Non-responders[b] (n=12)	P-value[c]
PTX3	D-0	-0.07 (-0.39 – 3.16)	-0.05 (-0.51 – 1.06)	0.68
	D+3	0.25 (-0.45 – 1.28)	-0.31 (-0.66 – 0.54)	0.08
	D+10	0.71 (-0.35 – 2.10)	-0.26 (-0.59 – 2.07)	0.03[§]
	D+30	0.13 (-0.73 – 0.70)	-0.26 (-0.79 – 4.24)	0.55
IL-8	D-0	0.02 (-0.61 – 0.14)	-0.11 (-0.92 – 2.92)	0.82
	D+3	-0.05 (-0.61 – 0.71)	-0.01 (-0.96 – 2.03)	1.00
	D+10	0.21 (-0.51 – 0.60)	-0.16 (-0.96 – 0.30)	0.04[§]
	D+30	-0.11 (-0.81 – 0.84)	-0.11 (-0.95 – 1.44)	0.89

Plasma cytokine levels of interleukin (IL)-8 and pentraxin (PTX)3 by ELISA on each day after UCB injection are shown as median value of their ratio to baseline (D-4), i.e., the differences from baseline to each time point per baseline, and their ranges (min. – max., parenthesis). [a]Responders refer to subgroups of group B (UCB + P-EPO), with more-improved GMPM changes during 12 months compared to the average GMPM change, whereas [b]Non-responders refer to those in group B with less-improved GMPM changes during 12 months compared to the average GMPM change. [c]P < .05, by Mann-Whitney U test.
